# Supplementary material for: Whole Genome DNA and RNA Sequencing of Whole Blood Elucidates the Genetic Architecture of Gene Expression Underlying a Wide Range of Diseases
Source: Res Sq. 2022 May 31:rs.3.rs-1598646. Preprint. [Version 1] doi: 10.21203/rs.3.rs-1598646/v1 (PMC9164515; doi:10.21203/rs.3.rs-1598646/v1)

Supplemental Materials

Whole Genome DNA and RNA Sequencing of Whole Blood Elucidates the Genetic Architecture of Gene Expression Underlying a Wide Range of Diseases

Chunyu Liu^1,2,#^, Roby Joehanes^3,#^, Jiantao Ma^4^, Yuxuan Wang^1^, Xianbang Sun^1^, Amena Keshawarz^3^, Meera Sooda^3^, Tianxiao Huan^5^, Shih-Jen Hwang^3^, Helena Bui^3^, Brandon Tejada^3^, Peter J. Munson^3^, Demirkale Cumhur^5^, Nancy L. Heard-Costa^2,6^, Achilleas N Pitsillides^1^, Gina M. Peloso^1^, Michael Feolo^5^, Nataliya Sharopova^5^, Ramachandran S. Vasan^2,6^, Daniel Levy^2,3,#^

^1^Department of Biostatistics, School of Public Health, Boston University, Boston, MA, USA

^2^Framingham Heart Study, Framingham, MA, USA

^3^Population Sciences Branch, Division of Intramural Research, National Heart, Lung, and Blood Institute, National Institutes of Health, Bethesda, MD, USA

^4^Nutrition Epidemiology and Data Science, Friedman School of Nutrition Science and Policy, Tufts University, Boston, MA, USA

^5^University of Massachusetts Medical School, Worcester, MA, USA

^6^Departments of Medicine and Epidemiology, Boston University Schools of Medicine and Public Health, Boston, MA, USA

**Supplemental Figure 1**.


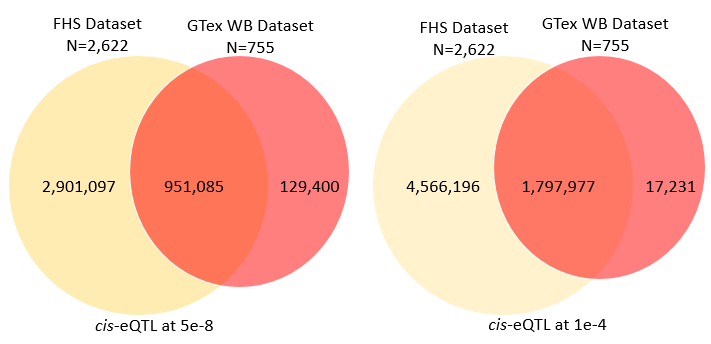

Supplement: Supplement 1 [file 5-12-22-eQTL_Supplemental_file_ScientificReport.docx]
